# Repeatability of a fully automated swept-source optical coherence tomography biometer and agreement with a low coherence reflectometry biometer

**DOI:** 10.1186/s40662-023-00343-4

**Published:** 2023-06-02

**Authors:** Alberto Domínguez-Vicent, Abinaya Priya Venkataraman, Amanda Dalin, Rune Brautaset, Robert Montés-Micó

**Affiliations:** 1 Division of Eye and Vision, Department of Clinical Neuroscience, Karolinska Institute, 171 77 Stockholm, Sweden; 2 Optics and Optometry and Vision Sciences Department, University of Valencia, Valencia, Spain

**Keywords:** Optical biometry, Swept-source optical coherence tomography, Low-coherence interferometry, Repeatability, Agreement

## Abstract

**Background:**

To evaluate the repeatability of a fully automated swept-source optical coherence tomography (SS-OCT) and its agreement with an optical low coherence reflectometry (OLCR) for several biometric parameters.

**Methods:**

In this study, 74 eyes of 74 patients were measured using the Eyestar 900 SS-OCT and Lenstar LS 900 OLCR. Flat keratometry (K1) and steep keratometry (K2), central corneal thickness (CCT), anterior chamber depth (ACD), lens thickness (LT), and axial length (AL) were measured three times with each device. The repeatability was analyzed with the intrasubject standard deviation, coefficient of variability (CoV), and coefficient of repeatability (CoR) for each instrument. The agreement between the instruments was evaluated with Bland-Altman analysis.

**Results:**

K1, K2 and CCT CoV values were < 0.2%, < 0.4% and < 0.55%, respectively. Higher CoV values were found for ACD and LT ranging from 0.56% to 1.74%. The lowest CoV values were found for the AL measurements (0.03% and 0.06% for the Eyestar 900 and the Lenstar LS 900, respectively). AL measurements provided the highest repeatability, measured with both CoV and CoR values, and the CCT was the parameter with the lowest repeatability. The CCT and LT measurements were statistically significant between the two biometers (*P* < 0.001). The interval of the limits of agreement was < 0.6 D for K1 and K2, 15.78 µm for CCT, 0.21 mm for ACD, 0.34 mm for LT, and 0.08 mm for AL.

**Conclusions:**

Both biometers provide repeatable measurements for the different parameters analyzed and can be used interchangeably.

## Background

The measurement of several ocular biometric parameters has become an essential procedure for ophthalmology and vision sciences. Detailed values of some parameters are mandatory for cataract and refractive surgeries but also for clinical diagnosis. In addition, due to the worldwide increase in myopia prevalence, the measurement of axial length (AL) becomes necessary to assess its progression. Taking into account the enormous applications in these fields there is a continuous development of new devices to measure ocular parameters.

The first attempt to measure ocular parameters such as AL or keratometry (K) using non-contact technology was done using partial coherence interferometry (PCI). This new technology is a better predictor of postoperative refraction than ultrasound biometry [1]. New technology has significantly improved the ability to accurately measure ocular biometrical parameters [2]. The improvements made over the last few years using different optical technologies based on PCI, optical low coherence reflectometry (OLCR), or swept-source optical coherence tomography (SS-OCT) have been high, making optical biometry an easier technique for measuring ocular parameters. Among the different optical technologies, optical biometers based on SS-OCT technology are likely to become the gold standard for ocular biometry [3]. This technology provides a deeper light penetration and long-range OCT imaging of posterior segment structures [4].

Validation studies for new optical biometers are needed to use them clinically in order to provide accurate measurements needed for intraocular lens (IOL) power calculation in cataract procedures but also for myopia progression evaluation. There is a new biometer available that is based on SS-OCT technology. Although this device has just been evaluated and compared with other biometers based on SS-OCT technology [5–7], to the best of our knowledge, there are no reports comparing this SS-OCT technology with OLCR. The purpose of this research was therefore to assess the repeatability and agreement of a SS-OCT and OLCR biometer.

## Methods

This prospective study was approved by the Regional Ethics Committee (Swedish Ethical Review Authority, No. 2021-03835) and was conducted in accordance with the tenets of the Declaration of Helsinki. Written informed consent was obtained from all patients prior to their enrollment in this study.

### Subjects and procedure

All the subjects participating in this study underwent a full ophthalmic examination. Inclusion criterion was healthy participants aged between 20 and 63 years old and exclusion criteria were participants with ocular trauma, severe corneal, crystalline lens or vitreous opacities, previous ocular surgery, diabetes, hypertension, retinal disease, glaucoma, or nystagmus. Flat keratometry (K1), steep keretometry (K2), central corneal thickness (CCT), anterior chamber depth (ACD, from corneal endothelium to anterior lens surface), lens thickness (LT), and AL were measured three times under repeatability conditions using the two optical biometers in a single session. Only one eye selected randomly from each patient was used for the data analysis. All the devices were calibrated prior to each measurement session, and the order of each instrument was randomized for each subject. Only scans with no missing areas and good coverage were considered acceptable.

### Optical biometers

Two optical biometers based on SS-OCT and OLCR techniques were analyzed in this study: the Eyestar 900 and the Lenstar LS 900 (both from Haag Streit AG, Koeniz, Switzerland). The Eyestar 900 (software version V2.2.0) is a fully automated biometer which performs automatic centration and measurement. It is a SS-OCT that uses a wavelength of 1060 nm with a scan speed of 30 kHz for AL measurements ranging from 14 to 38 mm. Dual zone keratometry is obtained using an infrared light-emitting diode (LED) source of 850 nm measuring 32 points. The Lenstar LS 900 (software version v.2.5.2) is a biometer that is based on OLCR and uses an 820 nm super luminescent diode to measure the CCT, ACD, LT, and AL. The K readings are calculated by analyzing the anterior corneal curvature at 32 reference points distributed in two concentric circles (16 points per circle) of approximately 2.30 and 1.65 mm diameters. A refractive index of 1.3375 was used for both instruments.

### Statistical analyses

Mean, standard deviation (SD), and range values were obtained for all the parameters measured. The data were entered into a Microsoft Excel spreadsheet (Microsoft Corp, Redmon, WA, USA). Repeatability and agreement were analyzed based on standards adopted by the British Standards Institute and the International Organization for Standardization [8]. Repeatability was assessed by calculating the following parameters: within-subject standard deviation (S_w_), coefficient of repeatability (CoR) and coefficient of variability (CoV). The CoR was expressed as a result of the SD of the difference between measurements ($\sqrt{2}\cdot {S}_{w}$). Thus, CoR was calculated as $1.96\sqrt{2}\cdot {S}_{w}$ and can be approximated as 2.77S_w_. The CoR represents the expected limits that 95% of the measurements should be within. The CoV was calculated as the ratio between S_w_ and the average value (x): CoV = ${S}_{w}/x$, and expressed in percentage. For a given parameter, the CoV quantifies the variation in the repeated measurements in relation to the average value of the parameter. The normality distribution was checked and confirmed by the Shapiro-Wilk’s test, and the difference between the paired measurements was evaluated with a t-test. The differences were considered significantly different when the *P* value was less than 0.05. The normality check and the paired comparison were analyzed using SPSS software (version 22.0, IBM Corp., USA). The agreement between both optical biometers was assessed using Bland-Altman analysis. The average difference, the confidence interval (CI) of the average difference at 95%, and 95% limits of agreement (LoA, calculated as mean difference ± 1.96 SD) were also ascertained.

The required sample size (n) was determined considering both repeatability and agreement. For repeatability, n can be calculated considering the number of repeated measurements (m) and confidence level (CL) for the estimated S_w_ as $\frac{1.96}{\sqrt{2n(m-1)}}$ = CL [9]. Considering a CL of 0.12 and 3 repeated measurements, 67 eyes are required. For agreement, the following formula was used, where s is the SD of the differences: 1.96 $\sqrt{\frac{{3s}^{3}}{n}}$ = desired CI of LoA. We considered the desired CI for the LoA in our study to be 0.01 mm for the AL (primary outcome). With this value and the s value obtained in a subset of 50 eyes, the minimum n value required was 59 eyes. Then, taking into account both n values, we considered that this should be at least 67 eyes, with our target being 70 eyes.

For further analysis of the keratometer values, the corneal astigmatism was analyzed using the double-angle plot. This plot presents the data points, centroid and 95% confidence ellipses [10].

## Results

In total, the study evaluated a total of 74 healthy eyes (36 right eyes and 38 left eyes) from 74 patients (55 females). The mean age of the patients was 31.0 ± 11.3 years (median: 26 years, range: 20 to 63 years). All eyes were measured using the two optical biometers. The mean spherical equivalent of the eyes analyzed was − 1.21 ± 2.32 D (mean ± SD), ranging from  − 8.50 to 2.38 D.

The repeatability outcomes of the two optical biometers for the different parameters analyzed are shown in Table 1. For K1, K2 and CCT, the S_w_ values were < 0.1 D, < 0.2 D and < 3 μm, respectively. The corresponding CoV values were < 0.2%, < 0.4% and < 0.55%, respectively. For ACD and LT, the S_w_ values were < 0.05 mm and < 0.07 mm, respectively, and this resulted in higher CoV values ranging from 0.56% to 1.74%. The S_w_ value for the AL measurements was lower than 0.02 mm, resulting in the smallest CoV values (0.03% and 0.06% for the SS-OCT based Eyestar 900 and the OCLR-based Lenstar LS 900, respectively). Taking into account all the ocular parameters, the AL measurement provided the highest repeatability (smallest S_w_ value), with the CCT being the parameter with the lowest repeatability (largest S_w_ value). In general, these values revealed that the repeatability of the Eyestar 900 and the Lenstar LS 900 to measure the different parameters analyzed was excellent.

**Table 1:** Repeatability analysis of the two optical biometers for the different parameters assessed

Parameter/Biometer	S_w_	CoV (%)	CoR
K1 (D)			
Eyestar 900	0.079	0.18	0.218
Lenstar LS 900	0.073	0.17	0.202
K2 (D)			
Eyestar 900	0.075	0.17	0.209
Lenstar LS 900	0.162	0.37	0.449
CCT (μm)			
Eyestar 900	1.559	0.29	4.320
Lenstar LS 900	2.936	0.54	8.133
ACD (mm)			
Eyestar 900	0.018	0.61	0.050
Lenstar LS 900	0.035	1.20	0.098
LT (mm)			
Eyestar 900	0.021	0.56	0.058
Lenstar LS 900	0.066	1.74	0.184
AL (mm)			
Eyestar 900	0.008	0.03	0.022
Lenstar LS 900	0.014	0.06	0.040

*K1* = flat keratometry; *K2* = steep keratometry; *CCT* = central corneal thickness; *ACD* = anterior chamber depth; *LT* = lens thickness; *AL* = axial length; *S*_*w*_ = within subject standard deviation; *CoV* = coefficient of variability; *CoR* = coefficient of repeatability

**Fig. 1:**
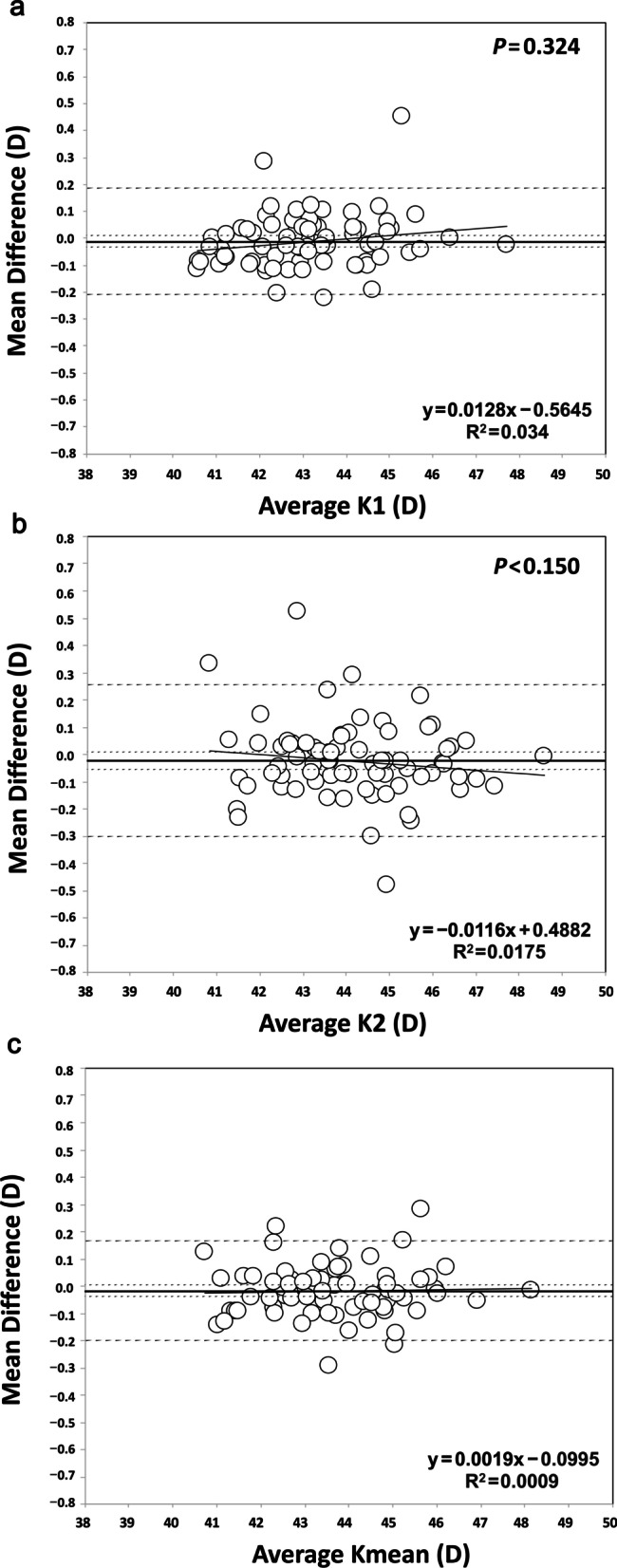
Bland-Altman plots showing the mean difference versus average of keratometry. **a** Flat keratometry (K1); **b** Steep keratometry (K2); **c** Mean keratometry (Kmean)

**Fig. 2:**
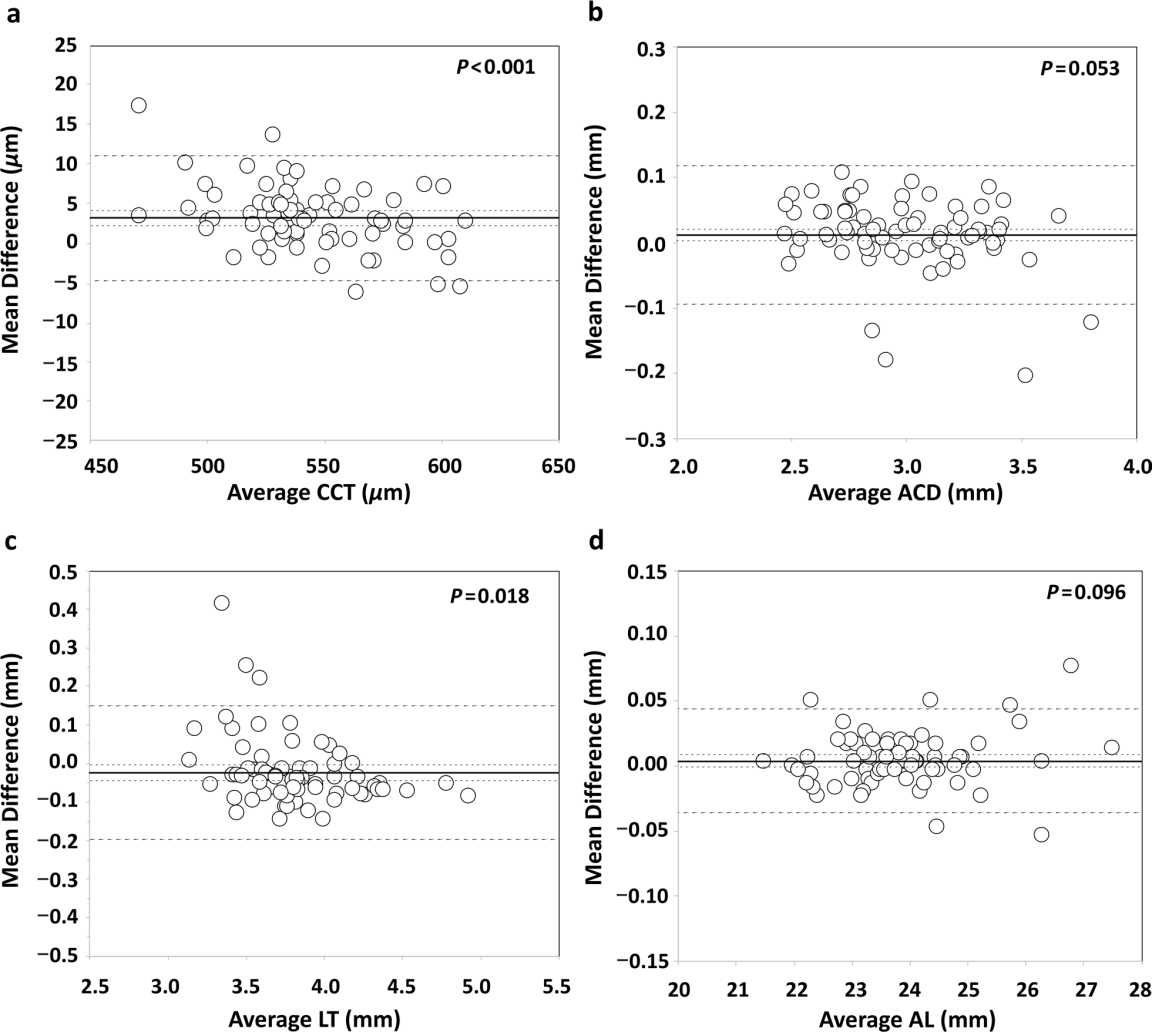
Bland-Altman plots showing the mean difference versus average of the (**a**) central corneal thickness (CCT), (**b**) anterior chamber depth (ACD), (**c**) lens thickness (LT) and (**d**) axial length (AL) for the comparison of the Eyestar 900 and the Lenstar LS 900 biometers. The mean (continuous line), lower and upper limits of agreement (± 1.96 SD [standard deviation], peripheral dotted lines), and the lower and upper confidence intervals (95%) are depicted. *P* values are included in each comparison

**Fig. 3:**
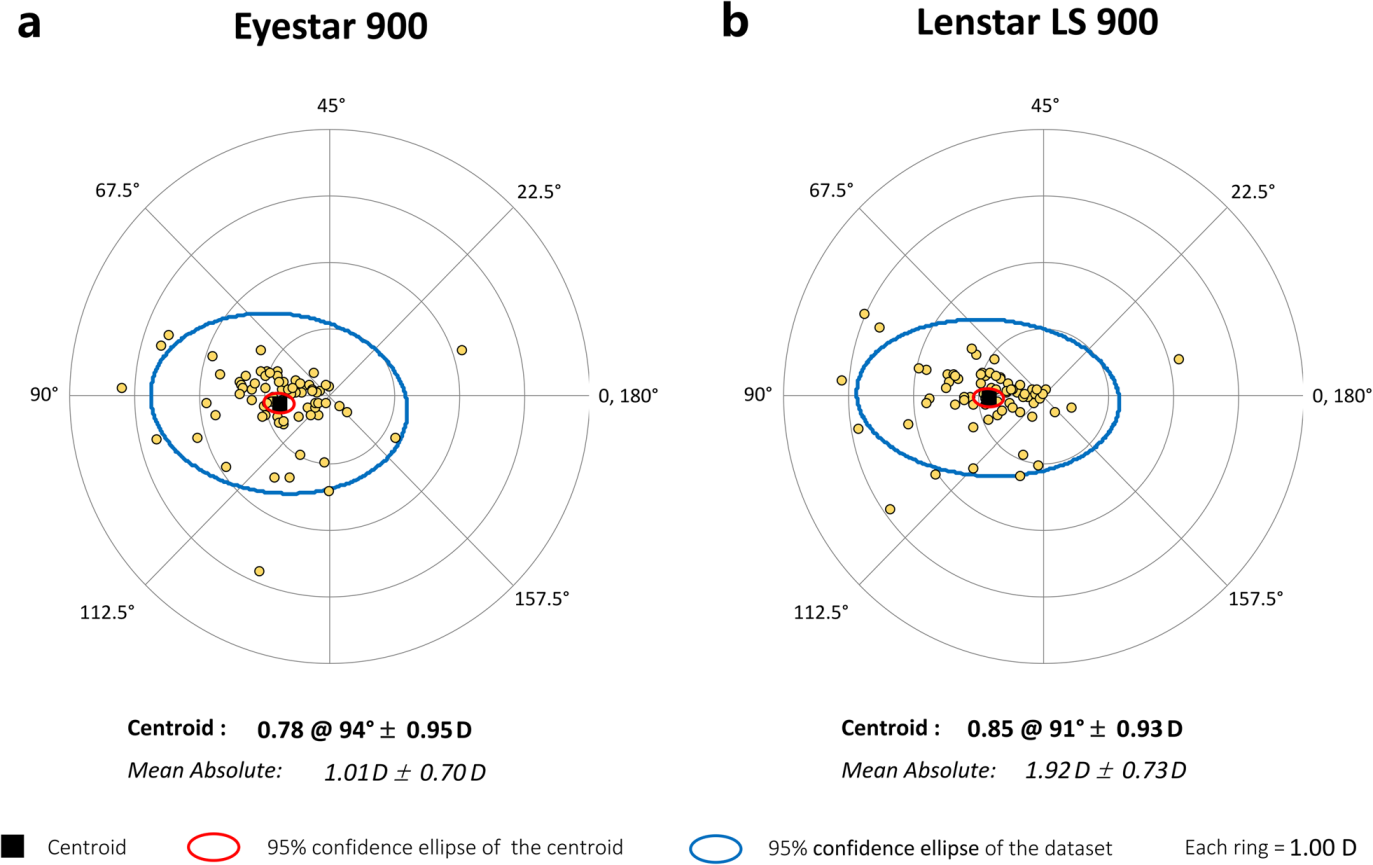
Double-angle plots for astigmatism were measured by the (**a**) Eyestar 900 swept-source optical coherence tomography (SS-OCT) and (**b**) the Lenstar LS 900 Optical Low Coherence Reflectometry (OLCR) biometers. These graphs show centroid and mean absolute values, the standard deviation and 95% confidence ellipses of the centroid and the dataset (each ring = 1.00 D)

Table 2 shows the mean measurement values, SD, and ranges for different parameters obtained using the two optical biometers. The outcomes for the agreement between the two optical biometers are shown in Table 3. For the comparison between both biometers, this table shows the mean difference ± SD, 95% CI, 95% LoA and LoA interval for the different parameters measured. T-tests revealed statistically significant differences between the two optical biometers for CCT and LT parameters evaluated (Table 2, *P* < 0.001 and 0.018, respectively). Figure 1 shows the Bland-Altman plots for K1, K2 and Kmean and Fig. 2 for CCT, ACD, LT and AL. Figure 3 shows a double-angle plot of the astigmatism measured with the two optical biometers, where the mean absolute and centroid values, and 95% of confidence ellipse of the centroid and the dataset are presented.

**Table 2:** Mean ± standard deviation measurement values (range) for the two optical biometers

Parameter	Eyestar 900	Lenstar LS 900	*P* value
K1 (D)	43.16 ± 1.47 (40.50–47.69)	43.17 ± 1.45 (40.62–47.71)	0.324
K2 (D)	44.17 ± 1.61 (41.01–48.58)	44.19 ± 1.63 (40.68–48.58)	0.150
CCT (µm)	546.59 ± 30.88 (472.67–611.67)	543.47 ± 32.43 (611.00–462.67)	< 0.001*
ACD (mm)	2.99 ± 0.31 (2.47–3.74)	2.98 ± 0.32 (2.45–3.87)	0.053
LT (mm)	3.80 ± 0.33 (3.15–4.89)	3.83 ± 4.97 (3.13–4.97)	0.018*
AL (mm)	23.98 ± 1.36 (21.48–28.98)	23.98 ± 1.36 (21.48–29.01)	0.096

*K1* = flat keratometry; *K2* = steep keratometry; *CCT* = central corneal thickness; *ACD* = anterior chamber depth; *LT* = lens thickness; *AL* = axial length; *P < 0.05

**Table 3:** Agreement between the two devices for different parameters analyzed

Parameter	Mean difference ± SD	95% CI	95% LoA	LoA interval
K1 (D)	− 0.011 ± 0.100	− 0.034, 0.011	− 0.208, 0.185	0.393
K2 (D)	− 0.024 ± 0.142	− 0.056, 0.008	− 0.302, 0.254	0.557
CCT (μm)	3.117 ± 4.026	2.199, 4.034	− 4.774, 11.008	15.783
ACD (mm)	0.012 ± 0.054	0.000, 0.024	− 0.093, 0.118	0.212
LT (mm)	− 0.024 ± 0.088	− 0.045, − 0.004	− 0.198, 0.148	0.347
AL (mm)	0.004 ± 0.020	− 0.001, 0.008	− 0.036, 0.044	0.080

*K1* = flat keratometry; *K2* = steep keratometry; *CCT* = central corneal thickness; *ACD* = anterior chamber depth; *LT* = lens thickness; *AL* = axial length; *SD* = standard deviation; *CI* = confidence interval; *LoA* = limits of agreement

## Discussion

The new fully automated Eyestar 900 uses SS-OCT technology to provide biometric measurements with the imaging of the anterior segment structures. The purpose of this study was to evaluate its repeatability and to evaluate the agreement with another well-used biometer, the Lenstar LS 900 (based on OLCR technology). The results obtained showed excellent repeatability (the CoV for all the parameters measured with Eyestar 900 and Lenstar LS 900 were lower than 1% and 2%, respectively) and good agreement between both instruments (the interval of the LoA was < 0.6 D for K1 and K2, 15.78 µm for CCT, 0.21 mm for ACD, 0.34 mm for LT, and 0.08 mm for AL.). To the best of our knowledge, this study is the first to compare the two devices.

The outcomes found in our study revealed that both instruments showed high levels of repeatability (Table 1). For the two biometers, AL is the parameter with the best repeatability (CoV: 0.03%–0.06% and CoR: 0.022–0.040 mm) while CCT was the parameter that had the lowest repeatability (CoR: 4.320–8.113 mm). There are numerous publications done using the Lenstar LS 900 on different type of eyes. Some of these publications agree with our results. Shammas and Hoffer [11] for example, evaluated 37 cataract eyes, reporting CoV values ranging from 0.001% to 0.006% for the different parameters analyzed in our study. These authors concluded that the precision of the measurements was very high. Specifically, in another study, Chen et al. [12] studied 40 eyes and found S_w_ values of 3.10 μm for CCT, 0.02 mm for ACD and 0.17 D for Kmean; and Zhao et al. [13] reported higher values of S_w_ for 56 myopic eyes (0.018 mm for AL, 0.052 mm for ACD, 0.181 D for K1, 0.301 D for K2 and 14.244 μm for CCT). In another group of 33 myopic eyes, Shen et al. [14] found S_w_ values of 0.016 mm for AL, 0.009 mm for ACD, 0.014 mm for LT and 1.982 μm for CCT, respectively. CoV values were small and varied from 0.3%–0.5%, except for AL, which was 0.06% (being in this case the same reported by us). McAlinden et al. [15] reported in 102 patients S_w_ (and CoR, in brackets) values of 0.02 (0.05) mm for AL, 0.02 (0.06) mm for ACD, 0.11 (0.29) D for K1 and 0.13 (0.36) D for K2. Ruiz-Mesa et al. [16] analyzed S_w_, CoR and CoV in 40 normal eyes, reporting higher values than us: 5.58 μm, 14.44 μm and 0.58%, for CCT; 0.04 mm, 0.11 mm, and 0.50%, for ACD; and 0.13 mm, 0.36 mm and 0.16%, for AL. The only other study reporting the repeatability of the Eyestar 900 [7], evaluated the repeatability of the Eyestar 900 in 56 eyes undergoing the preoperative work-up for cataract surgery or corneal refractive surgery and healthy volunteers. They found a good repeatability for the different parameters measured (AL, K, corneal astigmatism, CCT, corneal diameter, ACD, LT and lens tilting) with the CoV value less than 1% in most cases. They concluded that the Eyestar 900 produces highly repeatable measurements. Another study assessed the feasibility and repeatability of Lenstar in a large group of children and adolescents [17]. The results obtained in all these studies agree with those found in our series (Table 1).

In relation to the agreement between both devices, Table 2 shows the mean values and Table 3 shows the mean difference for each parameter evaluated. We found statistically significant differences for CCT and LT but not for other parameters. Figures 1 and 2 show the Bland-Altman plots. In relation to the K results we found mean differences < 0.1 D and 95% LoA interval < 0.6 D both for K1 and K2. The small differences in the agreement found in the present study for both K1, K2 and Kmean values suggest that the differences in the IOL power calculation using these two instruments would also be small. Despite the statistically significant differences between biometers for CCT, the mean difference was about 3 µm and the 95% LoA interval was around 16 µm. In relation to ACD, the mean difference was small (0.012 mm) but the 95% LoA interval was about 0.2 mm. We consider that this difference would not affect but should be taken into account when these biometers are used interchangeably. The mean difference for LT, which was statistically significant, was also small (− 0.024 mm) but the 95% LoA interval was large (0.347 mm). Thus, both biometers can be used interchangeably for LT measurements. Finally, with the AL measurements, our mean difference value was very small (0.004 mm) and also the 95% LoA interval (0.080 mm). If the repeatability of one or both of the instruments evaluated was not good, that could result in poor agreement between the instruments. In this study, we report that both instruments have good repeatability and agreement [18].

Since there are no publications comparing both technologies, some studies have several outcomes using different samples for repeatability and agreement with other SS-OCT biometers available in the market. For example, Sorkin et al. [5] have compared the Eyestar 900 with the Anterion SS-OCT (Heidelberg engineering, Germany) biometer in a sample of 133 eyes of 66 cataract patients (mean age of 71.6 ± 9.8 years and 62.1% females). They found that all differences were statistically significant except for differences in anterior K measurements (lower than 0.05 D) and no changes were noted with analysis considering only their right eyes. On average, the Eyestar 900 measured longer AL (0.014 mm), thicker CCT (7.1 μm), shallower ACD (0.031 mm) and thinner LT (0.127 mm) than the Anterion. The Bland-Altman analysis also showed excellent agreement for AL, ACD, CCT, anterior K1, anterior K2 and LT measurements. They reported a small consistent mean measurement bias in measurements of CCT (7.1 μm thicker in the Eyestar 900) and LT (0.13 mm thicker in the Anterion). These authors discussed that despite finding a mean difference of 0.014 mm in AL between both biometers, this difference would lead to a difference of roughly 0.05 D in IOL power calculation which can be considered clinically insignificant [19, 20]. They suggested that the measurement obtained can be considered clinically interchangeable between biometers. In another study, Lender et al. [6] evaluated three biometers in a sample of 157 eyes of 79 cataract patients (comparing the Eyestar 900 with the IOLMaster 700 [Zeiss, Germany]) and 38 eyes of 19 cataract patients (comparing the Anterion with the IOLMaster 700). They aimed to compare the different ocular parameters and assess the effect of possible differences found on the calculated IOL power for implantation in cataract surgery. Their results revealed that, when comparing the IOLMaster 700 to the Eyestar 900, no difference was found in AL, ACD, K1 or K2 measurements (*P* > 0.05). In contrast, AL and ACD measurements differed between the IOLMaster 700 and Anterion (*P* < 0.05), but not for K1 or K2 (*P* > 0.05). They indicated that the differences in measurements were found to be statistically significant but were minor enough to most likely be clinically insignificant. To establish interchangeability of the biometers, the Bland-Altman analysis indicated good agreement between all three biometers on most parameters, with a minor offset in ACD measurements between the IOLMaster and the Eyestar. In this study, in order to investigate whether the minor differences observed between the devices impacted the suggested IOL power, we input the mean output values of each device in several online formulae calculators and found that the calculated IOL power was 0.50–1.00 D lower with the IOLMaster 700. Finally, Galzignato et al. [7] evaluated the agreement between the Eyestar 900 and two other SS-OCTs: the IOLMaster 700 and the Argos (Inc, Santa Clara, CA). They found good to high agreement among the measurements of the three optical biometers, although some statistically significant differences were detected between the Eyestar 900 and the Argos (mean K, ACD, LT and corneal diameter were higher than the Argos). They also found that the Argos biometer measured a shorter AL in eyes > 25 mm. They concluded that the measurements obtained with the Eyestar 900 are in good agreement with those found with the SS-OCT and Argos devices.

Our study has some limitations, primarily due to including only healthy eyes. This information is useful for general comparisons, but future studies should also consider cataract patients and IOL power calculation for emmetropization. Second, it would have been interesting to include comparison with other optical biometers based on different optical technologies to ascertain possible differences among them. However, one of the most important strengths of our study was that we assessed the agreement with the OLCR-based Lenstar LS 900 biometer.

## Conclusions

In conclusion, our results demonstrate that the Eyestar 900 and Lenstar LS 900 provide repeatable measurements for the different parameters analyzed. Comparing the instruments, we believe that despite the statistically significant differences reported in CCT and LT and the LoA values, we consider them negligible from a clinical standpoint. Hence, the two biometers can be used interchangeably.

## Data Availability

All data supporting the findings of this study are available within the paper.
